# Inhaled nitric oxide as a rescue therapy in a preterm neonate with severe pulmonary hypertension: a case report

**DOI:** 10.1186/s13052-018-0494-9

**Published:** 2018-05-15

**Authors:** Martina Busè, Francesco Graziano, Fabio Lunetta, Giorgio Sulliotti, Vincenzo Duca

**Affiliations:** 1 Dipartimento di Scienze per la Promozione della Salute e Materno Infantile “Giuseppe D’Alessandro”, University of Palermo, Palermo, Italy; 2 Unità Operativa di Neonatologia e Terapia Intensiva Neonatale, AOOR Villa Sofia-Cervello, Palermo, Italy

**Keywords:** Inhaled nitric oxide, Preterm neonate, Pulmonary hypertension

## Abstract

**Background:**

Inhaled nitric oxide (iNO) has been approved for the treatment of persistent pulmonary hypertension of the newborn (PPHN) in term and near-term newborns. Its role in the management of persistent pulmonary hypertension in preterm infants is not clear. Although guidelines do not exist, some studies have shown that iNO could be used as a rescue therapy in preterm neonate with severe pulmonary hypertension.

**Case presentation:**

We describe the case of a preterm neonate, born at 30 + 1 weeks of gestation, with hypoxic respiratory failure not responding to maximal conventional therapy. On the third day of life echocardiography showed severe pulmonary hypertension with right to left shunt and therapy with iNO was started. We achieved a rapid improvement in clinical conditions and pulmonary pressure normalized after 42 h of treatment.

**Conclusions:**

Moving on a case by case basis, treatment with iNO should be considered as a rescue therapy in preterm newborns with acute hypoxic respiratory failure caused by severe pulmonary hypertension.

## Background

Persistent pulmonary hypertension of the newborn (PPHN) is a serious cardio-respiratory complication of the transition to extrauterine life. PPHN occurs mainly in term neonates, but it has also been detected in preterm neonates [1].

Inhaled nitric oxide (iNO) has been approved for the treatment of PPHN in term and near-term newborns. It was demonstrated that its selective vasodilatory action on the pulmonary circulation improves oxygenation and reduces the need for extracorporeal membrane oxygenation [2–7].

However, its role in the management of hypoxic respiratory failure due to pulmonary hypertension in preterm infants is not clear. Guidelines or standardized protocols do not exist, but some studies have shown that iNO could be used as a rescue therapy in preterm neonate with severe pulmonary hypertension who are not responding to maximal conventional therapy [8–13].

In the present paper, we describe the case of a preterm neonate, born at 30 + 1 weeks of gestation (WG), with severe pulmonary hypertension treated with inhaled nitric oxide.

## Case presentation

We report on the case of a female neonate born at 30 + 1 WG by emergency caesarean section for preterm premature rupture of membranes (PPROM). She is the first born of a bichorionic and biamniotic twin pregnancy. At born: Apgar score 1′ 5, 5′ 8, birth weight 1380 g. She first needed nasal continuous positive airway pressure (CPAP), followed by orotracheal intubation. A dose of surfactant was administered, then the neonate was immediately transferred to our Neonatal Intensive Care Unit (NICU).

Upon arrival to our NICU, the patient appeared suffering, hypotonic with reduced reactivity; oxygen saturation was 93–94% (inspired oxygen concentration (FiO2) 0.45) during synchronized intermittent positive pressure ventilation (SIPPV) and the abdomen appeared globose but treatable. She had emission of meconium with blood; blood and blood cores were suctioned from the oral cavity and stomach. Arterial blood gas analysis and routine investigations were normal. The coagulation was disrupted with prolonged prothrombin time and partial thromboplastin time and reduced antithrombin III activity (17%). This was corrected by administration of antithrombin III.

In the following hours the patient’s respiratory state deteriorated. Chest X-ray revealed the presence of pulmonary infiltrates, mainly to the left lung, and a second dose of surfactant was administered. Cranial ultrasound was normal. Echocardiography showed patent ductus arteriosus (PDA) and tricuspid insufficiency (PAPs 40–45 mmHg), but treatment was not started because of bleeding disorders.

On the second day of life echocardiography was repeated, showing PDA with pulsatile flow. The coagulation was normalized so we could start treatment with ibuprofen (first dose 10 mg/Kg). Broad-spectrum antibiotics were initiated.

**Fig. 1:**
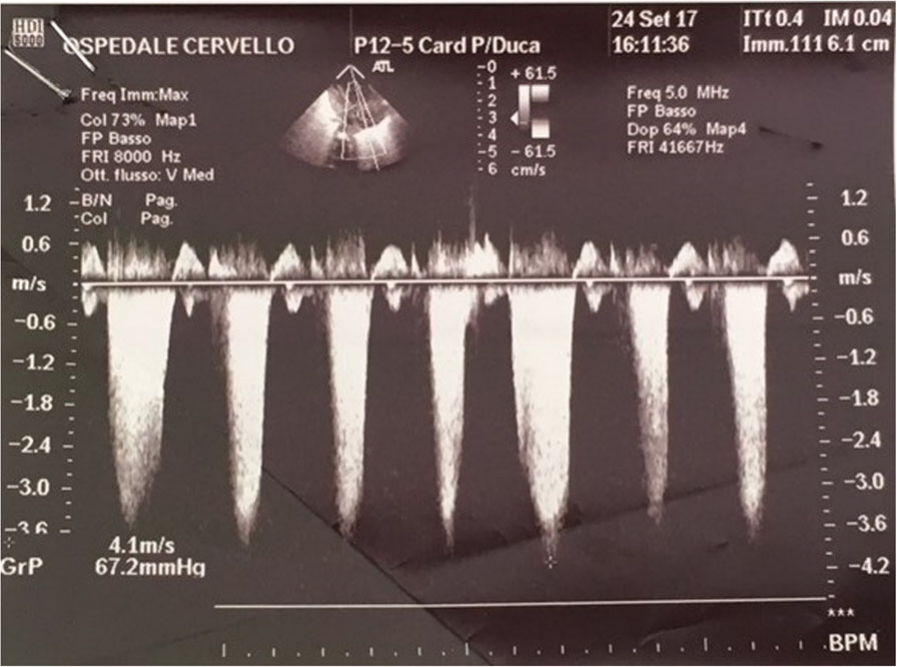
Doppler ultrasound showing severe pulmonary hypertension: velocity of tricuspid regurgitant jet up to 4.1 m/sec

On the third day of life the patient’s general conditions further deteriorated. Oxygen saturation was 80% during synchronized intermittent mandatory ventilation (SIMV) with FiO2 0.80–0.90. We observed a severe respiratory acidosis (pH 6.93, pCO2 75 mmHg, bicarbonate 11 mmol/l, base excess − 15.9 mmol/l). A third dose of surfactant and sodium bicarbonate were administered; simultaneously continuous dopamine infusion was started. The oxygenation index (OI), calculated as FiO2 x Mean Airway Pressure × 100/PaO2 (mmHg), was 16. Echocardiography showed severe pulmonary hypertension (PAPs 77–80 mmHg) with right to left shunt (Fig. 1). Given the critical condition of the infant and the finding of severe pulmonary hypertension, we decided to stop ibuprofen and start therapy with iNO. iNO was started at the dose of 10 p.p.m. and the neonate was ventilated in SIPPV (FiO2 0.95). After 1 h iNO was increased to 15 p.p.m. and conventional mechanical ventilation was switched to high frequency oscillation ventilation (HFOV). Eight hours after starting treatment, oxygen saturation and blood gas parameters were improving and we reduced iNO to 13, 10 and 7.5 p.p.m. gradually. At the same time conventional mechanical ventilation was restarted and FiO2 was progressively reduce to 0.50.

**Fig. 2:**
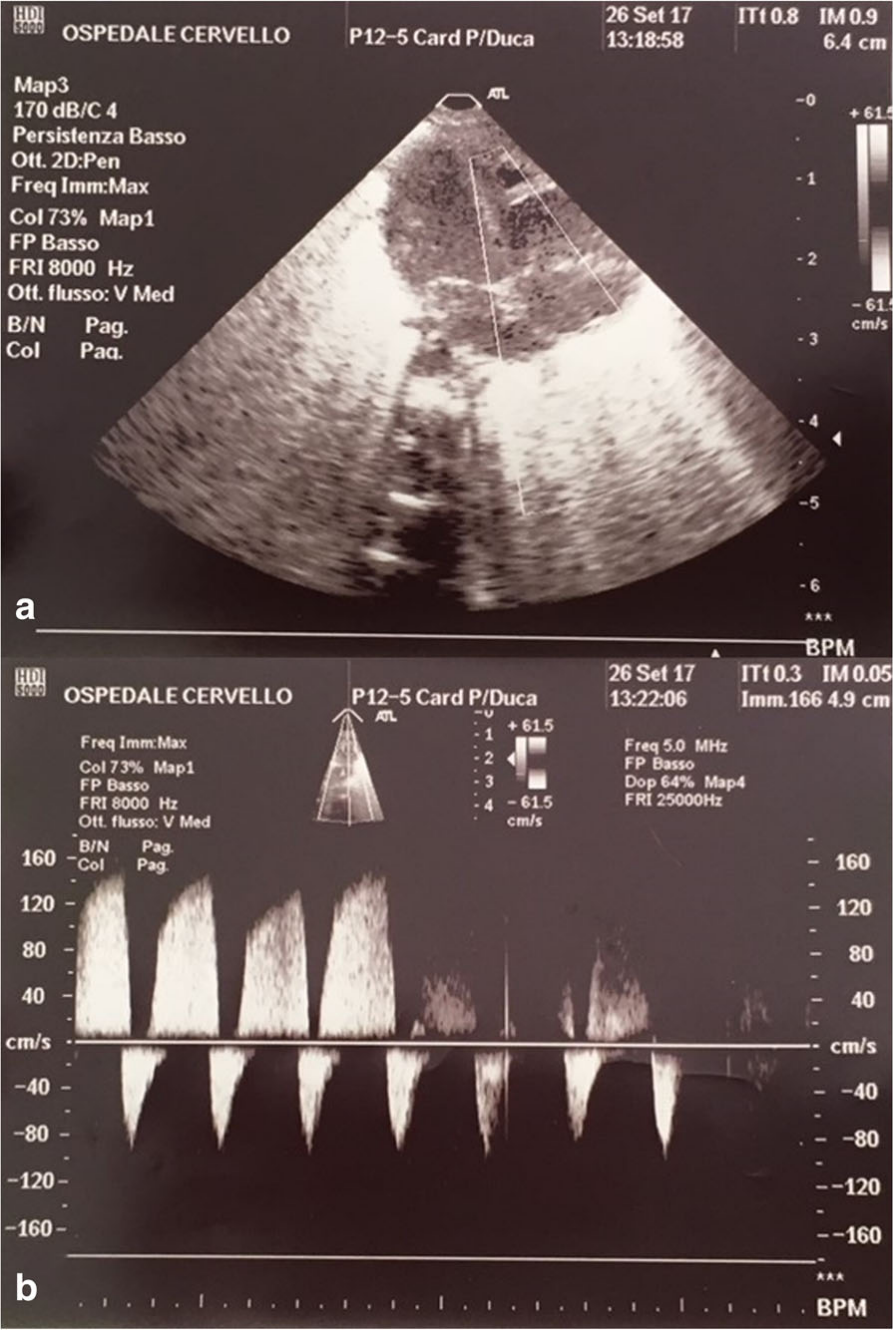
**a** Echocardiography showing prevalence of right heart chambers. **b** Doppler ultrasound: sisto-diastolic flow with almost totally left to right shunt

After 42 h of treatment echocardiography showed normal pulmonary pressure and PDA with almost totally left to right shunt (Fig. 2a and b). Oxygenation index was 9. iNO was progressively stopped, reaching the complete suspension after 50 h from the start of treatment. The patient continued conventional mechanical ventilation (SIMV), gradually lowering the FiO2, and at 60 h from iNO suspension she was extubated.

Cranial ultrasound performed at 32 + 5 WG was normal. Follow-up echocardiography was performed, the last (at 34 WG) showing normal pulmonary pressure, no shunts and closed ductus arteriosus.

## Discussion and conclusions

Although iNO has been approved for the treatment of PPHN in term and near-term newborns, the efficacy of this therapy for acute hypoxic respiratory failure owing to PPHN in premature neonates is not clear. Different studies have shown that iNO could be used as a rescue therapy in preterm neonates with severe pulmonary hypertension [8, 9]. In particular, it seems that the response to iNO improves significantly with increasing gestational age: neonates born ≥ 29 WG have a significantly greater response compared to neonates < 29 WG [10]. The most recent studies conclude that iNO therapy can improve the oxigenation in very preterm infants with PPHN, but it is not recommended for the routinely treatment and should be considered carefully. Moreover, they suggest that FiO2 > 0.65, echocardiographic diagnosis of PPHN, and birth weight > 750 g independently predict a beneficial effect of iNO in very preterm infants with RDS [12].

It is difficult to establish the cause of pulmonary hypertension in our patient, but it probably results from significant alteration of lung flows (with increased vasoconstrictive component) secondary to a maternal factors such as PPROM [10]. However, we cannot exclude that the administration of ibuprofen for PDA closure was the trigger that led to the onset of pulmonary hypertension. In fact, pulmonary hypertension is a rare but potentially lethal side effect in preterm infants receiving ibuprofen for PDA closure [14, 15].

After the finding of severe pulmonary hypertension, we started iNO therapy and we achieved a rapid improvement in clinical conditions. Treatment with iNO lasted a total of 50 h; the maximum dose reached was 15 p.p.m. During treatment the maximum value of methemoglobin was 1,4%. It is also important to note how the patient’s conditions improved when iNO has been associated with HFOV rather than with conventional mechanical ventilation, confirming the effectiveness of the association between iNO and HFOV for the treatment of PPHN.

Our NICU does not have a standard protocol regarding the use of iNO in preterm infants. At the discretion of the clinical team, and moving on a case by case basis, iNO therapy is used in preterm neonates with PPHN when even maximal conventional therapy (FiO2 0.80–0.90) is not working.

In conclusion, our experience confirms that treatment with iNO should be considered as a rescue therapy in preterm newborns with acute hypoxic respiratory failure caused by severe pulmonary hypertension. Further studies and clinical trials are needed to better determine the real efficacy of this therapy in preterm and extremely preterm infants and to define guidelines and standard protocols.

## Abbreviations

CPAP: Continuous positive airway pressure
FiO2: Inspired oxygen concentration
HFOV: High frequency oscillation ventilation
iNO: Inhaled nitric oxide
NICU: Neonatal intensive care unit
OI: Oxygenation index
PDA: Patent ductus arteriosus
PPHN: Persistent pulmonary hypertension of the newborn
PPROM: Preterm premature rupture of membranes
SIMV: Synchronized intermittent mandatory ventilation
SIPPV: Synchronized intermittent positive pressure ventilation
WG: Weeks of gestation
